# Positional distribution and conservation of major phosphorylated sites in the human kinome

**DOI:** 10.3389/fmolb.2025.1557835

**Published:** 2025-04-09

**Authors:** Athira Perunelly Gopalakrishnan, Prathik Basthikoppa Shivamurthy, Mukhtar Ahmed, Samseera Ummar, Poornima Ramesh, Sonet Daniel Thomas, Althaf Mahin, Mahammad Nisar, Sowmya Soman, Yashwanth Subbannayya, Rajesh Raju

**Affiliations:** ^1^ Centre for Integrative Omics Data Science (CIODS), Yenepoya (Deemed to be University), Mangalore, India; ^2^ Center for Systems Biology and Molecular Medicine (CSBMM), Yenepoya Research Centre, Yenepoya (Deemed to be University), Mangalore, India; ^3^ Department of Zoology, College of Science, King Saud University, Riyadh, Saudi Arabia; ^4^ School of Biosciences, Faculty of Health and Medical Sciences, University of Surrey, Guildford, United Kingdom

**Keywords:** predominant phosphorylated sites, kinome classification, kinase domain, kinase family, functional phosphorylated sites

## Abstract

The human protein kinome is a group of over 500 therapeutically relevant kinases. Exemplified by over 10,000 phosphorylated sites reported in global phosphoproteomes, kinases are also highly regulated by phosphorylation. Currently, 1008 phosphorylated sites in 273 kinases are associated with their regulation of activation/inhibition, and a few in 30 kinases are associated with altered activity. Phosphorylated sites in 196 kinases are related to other molecular functions such as localization and protein interactions. Over 8,000 phosphorylated sites, including all those in 517 kinases are unassigned to any functions. This imposes a significant bias and challenge for the effective analysis of global phosphoproteomics datasets. Hence, we derived a set of stably and frequently detected phosphorylated sites (representative phosphorylated sites) across diverse experimental conditions annotated in the PhosphoSitePlus database and presumed them to be relevant to the human kinase regulatory network. Analysis of these representative phosphorylated sites led to the classification of 449 kinases into four distinct categories (kinases with phosphorylated sites apportioned (PaKD) and enigmatic (PeKD), and those with predominantly within kinase domain (PiKD) and outside kinase domain (PoKD)). Knowledge-based functional analysis and sequence conservation across the family/subfamily identified phosphorylated sites unique to specific kinases that could contribute to their unique functions. This classification of representative kinase phosphorylated sites enhance our understanding of prioritized validation and provides a novel framework for targeted phosphorylated site enrichment approaches. Phosphorylated sites in kinases associated with dysregulation in diseases were frequently located outside the kinase domain, and suggesting their regulatory roles and opportunities for phosphorylated site-directed therapeutic approaches.

## Highlights

• Identification of predominantly detected phosphorylated sites in human kinases in global phosphoproteomes
• Characterization of kinases based on positional distribution of their predominantly detected phosphorylated sites
• Over 50% of the predominantly detected phosphorylated sites in Serine/Threonine kinases were found outside their kinase domain, and in Tyrosine kinases, they were distributed both within and outside the kinase domain
• Identification of conserved and non-conserved phosphorylated sites in kinases within kinase families and unique kinase-specific pathways

## Introduction

Phosphorylation is one of the major post-translational modifications (PTMs) in proteins and are catalyzed by enzymes known as kinases. The Human genome encodes over 500 kinases including 478 eukaryote-kinases (ePK), 40 atypical kinases (aPK) and 106 pseudo-kinase and represents one of the largest functionally diverse groups of proteins (Manning et al., 2002; Caenepeel et al., 2004; Hanks, 2003). Phosphorylation is established as a mode of regulation of the turnover and enzymatic activity along the molecular functions such as protein-protein interactions and subcellular localizations of substrate proteins, including those in kinases (Acconcia et al., 2007; Yogosawa and Yoshida, 2018; Hossain et al., 2016; Iyer et al., 2020; Guo et al., 2016; Lu and Zhou, 2007; Tarrant and Cole, 2009). The phosphorylation of specific sites within kinases is entailed by homomeric interactions often referred to as autophosphorylation and/or heteromeric interactions between kinases across kinase families (Zhang et al., 2006; Rauch et al., 2011; Keshava Prasad et al., 2009). Such kinase-kinase regulatory phospho-signaling networks are pivotal in cell signaling to direct biological processes and cell fate. Consequently, dysregulation of expression or activity of one or more protein kinases contributes to the initiation and progression of various disorders including cancers (Chou et al., 2020; Hanahan and Weinberg, 2011), developmental (Ng et al., 2019), neurological (Lassen et al., 2017), and immune disorders (Pike and Tremblay, 2018; Wang and Cole, 2014; Harsha and Pandey, 2010; Lahiry et al., 2010; Xu et al., 2019). Evidently, kinases are of high therapeutic relevance, and a large number of kinase inhibitors are explored till date (Scott et al., 2023).

The activation or inhibition of many kinases relies on specific phosphorylation sites, which are integral to the phospho-regulatory signaling network that orchestrates biological processes and are frequently examined as markers of kinase regulation. Hence, the identification of specific phosphorylated sites in kinases that are predominantly associated with their regulation and function is crucial for the progressive understanding of the cell signaling network (Manning et al., 2002; Lander et al., 2001). However, the corresponding experimental validation is often limited to the kinases with the availability of antibodies specific to those phosphorylated sites. In this context, there is a continuous quest to unveil the role of relevant phosphorylation sites in kinases and their substrates in specific biological conditions and/or cell types. Consequent to an outburst of global phosphoproteomics datasets in the public domain, a large number of phosphorylated sites in kinases are reported in diverse cell types and tissues across distinct physiological or pathological experimental conditions (Sriraja et al., 2023; Al Tarrass et al., 2024).

It is observed in multiple global phosphoproteome datasets that certain phosphorylated sites in a kinase are frequently detected (Xiao et al., 2022). As an example, the Y419 within the kinase domain of proto-oncogene tyrosine-protein kinase Src (SRC) is an established activation-associated phosphorylated site most studied through low-throughput approaches. Among the 39 phosphorylated sites reported, Y419 represented SRC in almost 36% of the total global phosphoproteome datasets, while Y530, its second most detected activation-associated phosphorylated site, represented SRC in 12% of datasets (Roskoski, 2005; Hornbeck et al., 2019). However, such representatively/predominantly detected phosphorylated sites in specific kinases and their efficient positioning in a manner that can contribute to a prospective analysis of global phosphoproteomes is not currently envisaged.

Phosphorylated sites in kinases that are distant from the active site, entailed as allosteric phosphorylated sites, either within or outside the kinase domain, can also induce conformational changes. This could be relatively better visualized with respect to activation-inducing or other structurally relevant mutations in kinases (Wu et al., 2014). Such phosphorylation events may result in the modulation of the kinase activity or alter the accessibility or enhanced affinity to specific substrates (Godbole and Dokholyan, 2023). For instance, the auto-inhibitory S9 phosphopeptide in GSK-3β at N-terminal region outside its kinase domain binds to the substrate-binding pocket as a pseudo-substrate and stabilizes a conformation that inhibits GSK-3β kinase activity (Stamos et al., 2014). Phosphorylation of sites outside the kinase domains have also been shown to affect the kinase stability and half-life. As an example, the phosphorylation together at T638 and S657 in the C-terminal region of protein kinase C-alpha (PKCA) have been associated to enhanced stability and maturation, while mTORC2-mediated phosphorylation at S657 prevents its degradation (Aslam and Alvi, 2022; Ikenoue et al., 2008). Furthermore, phosphorylation at C-terminal S473, S477, and T479 residues in AKT1 (Liu et al., 2014) and at T347 in CSNK1D outside their characteristic kinase domain are established to induce their enzymatic activity (Eng et al., 2017), whereas autophosphorylation of CSNK1E at S405, T407, and S408 within its kinase domain is associated with the inhibition of its activity (Liu et al., 2002). Such phosphorylated sites with known functions in kinases are also frequently observed in phosphoproteomes and contribute to its robustness to explore the predominant phosphorylated sites being reported in them. In this context, a large number of phosphorylated sites seemingly without any known functions over-represent many kinases and are often ignored in the analysis of phosphoproteome datasets (Priyanka et al., 2024; Sanjeev et al., 2024a; John et al., 2024; Sanjeev et al., 2024b).

Multiple tools such as Kinase Enrichment Analysis (KEA) (Lachmann and Ma’ayan, 2009), Kinase Enrichment Analysis 3 (KEA3) (Kuleshov et al., 2021) Kinase-Substrate Enrichment Analysis (KSEA) (Wiredja et al., 2017), PTM-Signature Enrichment Analysis (PTM-SEA) (Krug et al., 2019), Integrative Inferred Kinase Activity (INKA) (Beekhof et al., 2019), Phosphorylation Set Enrichment Analysis (PSEA) (Suo et al., 2014), Inference of kinase activities from phosphoproteomics (IKAP) (Mischnik et al., 2016) etc*.*, are employed for the functional enrichment analysis of global phosphoproteome datasets. Intriguingly, INKA encompasses phosphorylated sites located within kinase domains and considers them to be directly associated with kinase activity (Beekhof et al., 2019). However, this would exclude a large number of frequently detected regulatory phosphorylated sites located outside the kinase domain. Phosphorylated sites outside the kinase domain are often found in pseudo kinase domains, hinge regions, or disordered regions as shown in the examples above may entail critical roles in modulating kinase activity, specificity to substrate interactions, and functional complex formation (Needham et al., 2019).

**FIGURE 1:**
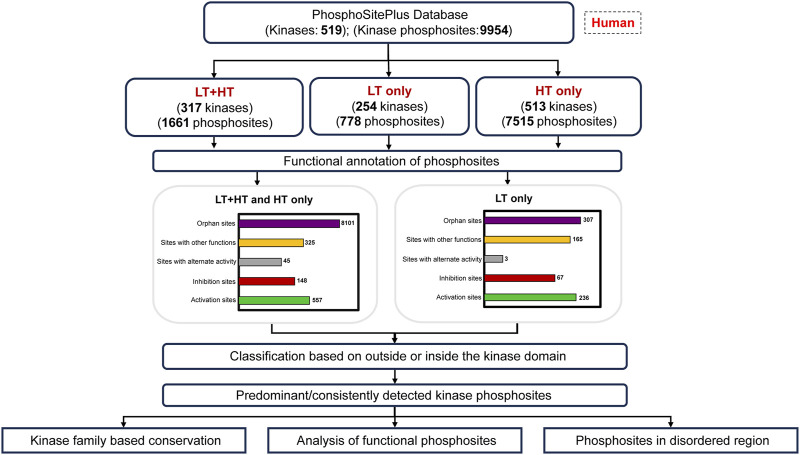
Workflow for the analysis of predominant phosphorylated sites and the categorization of human kinome. LT: Datasets from Low Throughput experiments; HT: Datasets from High Throughput experiments.

The kinases are currently classified based on diverse sequence features such as sequence similarity, evolutionary lineage, domain composition, etc*.* (Manning et al., 2002; Martin et al., 2010; Cohen, 2002). Predominantly detected phosphorylated sites in proteins irrespective of their association with activity or other specific functions are likely to represent the kinase as its major phospho-proteoform in global phosphoproteomes. Lack of visualization or information on many of them has created a critical gap in the effective interpretation and progressive analysis of the phosphoproteomics datasets. A characterization of phosphorylated sites that predominantly represent the kinases will essentially prioritize the assessment of their functional roles and subsequently, enhance research towards their selective therapeutic strategies (Xiao et al., 2022). Furthermore, representative kinase phosphorylated sites can be engaged as a platform for intuitive hypothesis-driven analysis of their kinome regulatory networks. Hence, we propose a contemporary phosphoproteome based classification of kinases accounting for their representative phosphorylated sites. Although other PTMs, such as acetylation, methylation, and glycosylation, etc., also contribute the accessibility of phosphorylation sites and the activity of kinases and phosphatases, predominant phosphorylated sites in specific kinases can provide a framework to accelerate research on their cross-talk with other PTMs in the future (Csizmok and Forman-Kay, 2018). In our efforts to address these gaps, we classified human kinome based on multiple functional features of the predominant phosphorylated sites in kinases as represented in the workflow (Figure 1).

## Materials and methods

### Assembly of phosphorylated sites in the human kinome

The list of 537 characterized human kinases was retrieved from KinHub (Eid et al., 2017). These kinases included those with typical kinase domains and atypical kinases characterized based on Manning et al. (2002). Phosphorylated sites reported in these human kinases were obtained from the PhosphoSitePlus database, a comprehensive well-annotated resource of post-translational modifications (PTMs), especially on the phosphorylated sites. The sites that are uniquely reported from unpublished CST datasets were excluded from further analysis. The phosphorylated sites that are detected/validated in one or more mass spectrometry-based phosphoproteomics experiments, referred to as high-throughput sites (HTP), or studied using targeted phosphorylated site-specific antibodies or site-centric mutation analysis, referred to as low-throughput sites (LTP), were considered for the current analysis.

To analyse the mass spectrometry-based detectability for the LTP-specific phosphorylated sites, performed an in silico trypsin digestion and filtered the peptides containing phosphorylated sites. Based on the number of amino acids contained in each peptide, categorized into the peptides with 8–15 amino acids and greater than 15 or less than eight amino acids groups.

### Extraction of predominant phosphorylated sites in human kinases

Phosphorylated sites in kinases were ranked based on the frequency of their detection in diverse HTP datasets and the number of their targeted studies supported by LTP experiments. The top ten phosphorylated sites with the highest frequency of detection/assignment in HTP experimental datasets were considered and tagged as the most predominantly detected phosphorylated sites. Each of these predominant phosphorylated sites were further analyzed for their association with the activation or inhibition status of kinases, or their association with other functions such as molecular regulation, intracellular localization, protein stabilization, protein degradation, and protein conformation. The regulatory site information annotated in PhosphoSitePlus was engaged for this association. The phosphorylated sites that are currently not associated with any of these functions were categorized as “orphan” sites (Supplementary Table S1).

### Analysis of kinase family-based conservation of predominant phosphorylated sites

The evolutionary conservation of phosphorylated sites within and across kinase families was analyzed based on protein sequence parameters. A conservation score was generated for each reported phosphorylated site. The score accounted for factors including sequence similarity, property matching, gap penalty, etc, Our methodology centered on extracting window sequences of 11 amino acids including the central phospho-acceptor site (p) and computing conservation scores across kinase family-aligned sequences. Using Biopython’s SeqRecord objects, kinase sequences were curated in FASTA format, and ClustalW alignment was followed to structurally organize these sequences. This facilitated the discovery of conserved regions that may indicate evolutionary relationships and functional correlations essential to kinase activity. Post-alignment, the Biopython AlignIO module enabled targeted feature extraction from these aligned sequences, supporting a kinase family-based scoring system.

Given a phosphorylated site position p within a sequence of length L, we extracted a window of size 2*w* + 1, where w is the window size parameter which is five, resulting in a total window length of 2 × *w* + 1 = 11.

Let:

*S* represents the aligned sequence.

Wp be the window centered at $p:W_{p}=S\left[p-w:p+w\right].$

If the window is shorter than 11 due to sequence boundaries, padding with gaps (‘*-*’) is applied.

#### Scoring Criteria

For each alignment position corresponding to the extracted window, a score is calculated based on matches and mismatches to the central phosphorylated site and surrounding residues. Each amino acid residue in the window at position *j* (relative to *p*) is compared to the aligned residues at the same position in other sequences.

Let:

$R_{p+j}$ be the residue in the window $W_{p}$ at relative position j from the phosphorylated site.

$A_{p+j}=\left\{a_{1},a_{2},\ldots .,a_{n}\right\}$ be the set of residues from the aligned sequences at position p + j.

We have defined the main scoring components such as exact matches, property matches, and gap penalties.

Exact Matches: Count for residues $a_{i}$ in $A_{p+j}$ where $a_{i}=R_{p+j}$

Property matches: The count of residues $a_{i}$ in in $A_{p+j}$ where $a_{i}=R_{p+j}$ has a similar property, based on a predefined similarity dictionary.

Gap penalties: Number of gaps (‘-’) in $A_{p+j}$

Based on these defined functions, we calculated the score contribution for each position. To keep the score directly in the range of 0–1, we adjusted the scoring system to assign the maximum possible score for each position within the window, achieving a bounded score. Each position in the window will contribute a maximum score of 1, divided between exact matches, property matches, and gaps. This approach ensures that each residue contributes proportionally within a range of 0–1.

$$\text{score}\left(p+j\right)=\frac{\text{exact}\_\text{matches}\left(p+j\right)}{n}\times w_{\text{exact}\;}+\frac{\text{property}\_\text{matches}\;\left(p+j\right)}{n}\times w_{\text{property}\;}+\frac{\text{gaps}\;\left(p+j\right)}{n}\times w_{\text{gap}}$$

The final score across the entire window (centered at p) is calculated based on the sum of the scores for each position:

$$\text{total}\_\text{score}=\sum_{j=-w}^{w}\text{score }\left(p+j\right)$$

Each residue contribution is bounded by its match quality and penalized for gaps, allowing the score to reflect both conservation and similarity in the context of the alignment window.

### Analysis of the location of the predominant phosphorylated sites relative to the kinase domain

To examine the positional distribution of the predominant phosphorylated sites in each kinase, phosphorylated sites were assessed relative to their protein kinase domains derived from the InterPro database (Paysan-Lafosse et al., 2023). We used in-house Python scripts to determine whether each phosphorylated site is located within or outside the kinase domains. The occurrence of the predominant phosphorylated sites in regions in kinases currently classified as disordered regions was also analyzed based on the Database of Disordered Protein Prediction (D2P2) database (Oates et al., 2013). Kinase missense mutations associated with cancers were retrieved from the database KinaseMD (kinase mutations and drug response) (Hu et al., 2021). Considering the positional distribution of the top three phosphorylated sites, kinases were categorized as Predominant phosphorylated site in the Kinase domain (PiKD), Predominant phosphorylated site outside the Kinase domain (PoKD), Phosphorylated sites mixed in- and outside Kinase domain is represented as Phosphorylated sites apportioned in Kinase and distributed (PaKD), and Predominant phosphorylated sites enigmatic to the Kinase and kinase domain (PeKD).

### Inferring functional phosphorylated sites in predominant kinase phosphorylated sites

Using the functional score proposed by Ochoa et al. (2020), we have analyzed functional phosphorylated sites among these predominant phosphorylated sites. Ochoa et al. predicted the functional score based on different features, including sequence characteristics, domain localization, and conservation profiles trained on a machine learning model, aiming to predict functional relevance for lesser-characterized phosphorylated sites in the kinome.

## Results

### A landscape of phosphorylated sites in the human kinome

Over the past decade, the advent of mass spectrometry- and other array-based platforms for the analysis of PTMs have transformed our focus on the gene-centric function of proteins into PTM-centric functions. It is well-established that the PTMs are critical determinants of the regulation of turnover, localization, and functions of proteins. Currently, exemplifying the role of kinases, around 17,000 proteins are reported to be phosphorylated in humans (Hornbeck et al., 2019). The 537 kinases characterized in the human genome are also functionally accounted for by their array of phosphoproteoforms. The characterization of these phosphorylated sites or the phosphoforms is inevitable for our better understanding of the human kinome regulatory networks through progressive analysis of the phosphoproteomes.

**FIGURE 2:**
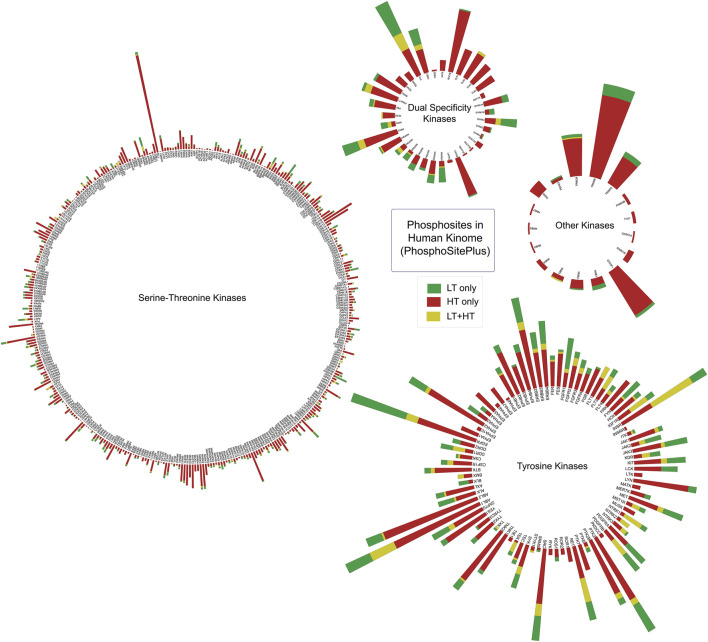
Ranking serine/threonine, tyrosine, and dual-specific kinases based on the number of phosphorylated sites extracted from PhosphoSitePlus database. The height of each bar represents the total number of phosphorylated sites identified in a kinase. Number of phosphorylated sites identified in each source of experiments were represented in different colours. LT: Datasets from Low Throughput experiments; HT: Datasets from High Throughput experiments.

The literature-curated experimentally-verified information on phosphorylated sites reported using low-throughput and/or high-throughput approaches including those by mass spectrometry were obtained from the PhosphoSitePlus database (Hornbeck et al., 2019). Together, 9,954 experimentally-verified phosphorylated sites reported in 519 human protein kinases were compiled. It includes 380 Serine/Threonine kinases, 87 Tyrosine kinases, 35 dual specificity kinases and 17 other types of kinases (Supplementary Table S1; Figure 2).

### Characterization of kinome phosphorylated sites based on their contemporary validation

Phosphorylated site identification relies on both low-throughput (LTP) and high-throughput (HTP) methods, each offering distinct advantages that contribute to a comprehensive understanding of kinase phosphorylation landscape across phosphoproteomics datasets from diverse experimental contexts and disease conditions (Hornbeck et al., 2019). Low-throughput methods for identifying phosphorylated sites typically involve targeted, small-scale experiments such as site-directed mutagenesis, Western blotting, or kinase assays, which provide high specificity and confirmation of individual phosphorylation sites. In contrast, high-throughput methods, such as mass spectrometry-based phosphoproteomics, enable large-scale identification of thousands of phosphorylated sites across multiple kinases simultaneously, providing a broader view of phosphorylation landscapes. While LT methods offer high confidence in site validation, HT methods are invaluable for discovering novel phosphorylated sites and mapping kinase signaling networks, making both approaches complementary for comprehensive phosphorylated site characterization.

Our analysis reveals that just 17% of the total phosphorylated sites are validated, while approximately 75% remain unvalidated in mass spectrometry-based studies. Furthermore, 8% (778 sites) were detected solely through LT approaches, lacking validation from HT or mass spectrometry methods. We speculated that this limitation in detection may be attributed to the peptide length and proteolysis protocol constraints of mass spectrometry, which optimally identifies proteolytic enzyme-specific peptides predominantly within 8–15 amino acids or 500–2000 Da in mass (Fricker, 2015; Swaney et al., 2010).

To explore this, we conducted an *in silico* digestion of proteins specific to the LT-only group. Most phosphopeptides in this group fell outside the 8–15 amino acid peptide range. However, 28% (233/819) of digested peptides spanned between 8 and 15 amino acids and their lack of detection indicates low ionization efficiency, phosphate group instability, charge state, fragmentation behaviour, *etc.* (Orsburn et al., 2011; Gafken and Lampe, 2006; Knight et al., 2003). Approximately 72% of peptides were either shorter than eight amino acids or exceeded 15 amino acids in length which might contribute to their lower detection rates by mass spectrometry (Supplementary Figure S1).

To classify the phosphorylated sites based on experimental validation, we categorized them into three groups: (i) sites with both low-throughput (LT) and high-throughput (HT) evidence (LT + HT), (ii) sites identified exclusively through HT approaches (HT), and (iii) sites identified only via LT studies (LT). The LT + HT group encompasses 1,661 phosphorylated sites from 317 kinases. Meanwhile, the HT category comprises 7,515 phosphorylated sites from 513 kinases, and the LT group contains 778 phosphorylated sites from 254 kinases. For the phosphorylated sites encompassed in the PhosphoSitePlus database, we ranked each phosphorylated site based on the frequency of detection in these datasets. The phosphorylated sites most frequently detected in specific kinases were considered as their predominant phosphorylated site. (Figure 2; Supplementary Table S1).

### Conservation of predominantly detected phosphorylated sites across the kinase family

Analysis of conserved and unique phosphorylated sites across kinase families provide key regulatory insights that enhance our understanding of kinase function, specialization, and therapeutic potential. Conserved phosphorylated sites appear to underpin core regulatory functions shared across kinases, including activation, substrate specificity, and protein interactions, indicating that these sites are essential for maintaining fundamental kinase activity and functionality within the family. Unique phosphorylated sites, on the other hand, suggest functional divergence and specialization among kinases, reflecting adaptation to specific cellular contexts or distinct signaling pathways. This dual characterization of phosphorylated sites offer valuable guidance for drug development strategies: conserved sites present opportunities for broad-spectrum kinase inhibitors, while unique sites enable selective targeting, potentially reducing off-target effects. Furthermore, the conservation and uniqueness of these phosphorylated sites reveal evolutionary and structural adaptations in kinase domains, where conserved sites are likely crucial for structural stability, and unique sites may highlight regions adapted for specialized interactions. Importantly, this analysis also aids in understanding kinase-related disease mechanisms, as mutations or dysregulation in conserved sites can have broad pathological implications, while unique site alterations may drive kinase-specific disease phenotypes. Together, these findings provide a nuanced perspective on kinase biology, regulatory diversity, and therapeutic targeting, underscoring the value of examining conserved and unique phosphorylated sites in elucidating kinase family functions and disease associations.

**FIGURE 3:**
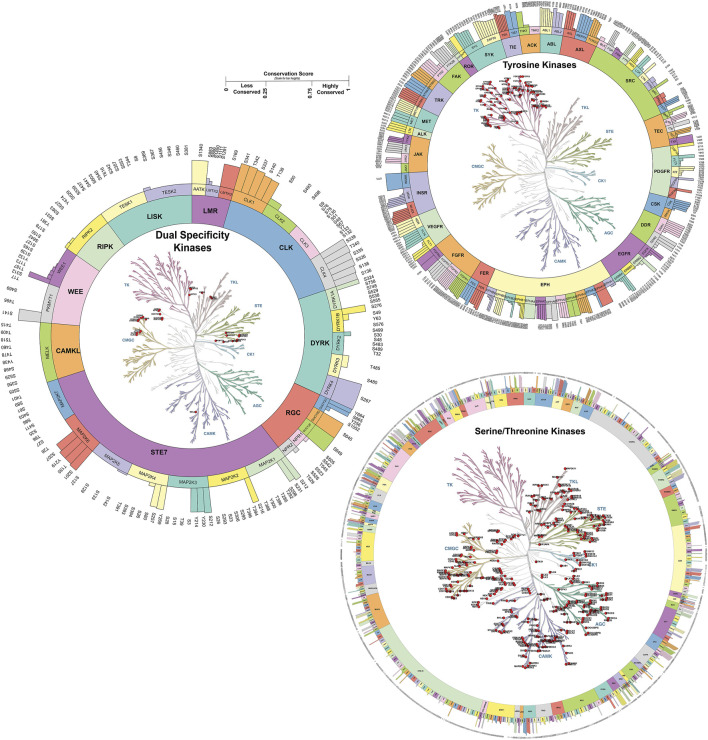
Phosphorylated site conservation across the kinase family for Serine/Threonine, Tyrosine, and Dual specificity kinases. The conservation score ranges from 0 to 1. Highly conserved sites with a score of one and sites with a score of zero were considered to have less conservation across the family. The conservation score was generated based on sequence similarity properties. Kinase tree generated using KinMap tool.

We analyzed the conservation of predominant phosphorylated sites across kinase families, identifying both conserved and unique sites. Kinase families with only a single member were excluded from this analysis. In total, 82 kinase families were examined, comprising 2,624 predominant phosphorylated sites. A conservation score, ranging from 0 to 1, was calculated based on sequence alignment and other features such as residue propensity. Phosphorylated sites with a score of ≥0.75 were classified as highly conserved, while those scoring below or equal to 0.25 were classified as less conserved (Supplementary Table S2). Our analysis revealed that 290 kinases contained both conserved and non-conserved phosphorylated sites (2639 sites). We have analyzed less conserved sites and identified 1528 unique phosphorylated sites with less conservation across the respective kinase family (conservation score <0.1) (Figure 3). Less conserved phosphorylated sites across kinase families can contribute to the opportunities for selective therapeutic targeting, enabling precise inhibition of specific kinases with minimal off-target effects. They also offer insights into functional specialization, allowing researchers to study pathway-specific roles of kinases, and serve as potential biomarkers for disease-specific alterations, aiding in diagnosis and monitoring of kinase-related conditions.

To gain deeper insights into these less conserved phosphorylated sites and their interactions, we analyzed specific kinase-kinase interactions that can be considered unique pathway reactions, focusing on upstream kinases that have been experimentally validated to phosphorylate distinct phosphorylated sites within specific kinase families. This analysis led to the identification of numerous kinase-substrate interactions that are exclusive to particular members within a kinase family (Supplementary Figure S2). Additionally, we identified several phosphorylated sites in 20 kinases that serve as unique auto-phosphorylation sites, along with 99 heteromeric kinase-kinase interactions specific to these uniquely phosphorylated sites (Supplementary Table S2). For instance, PAK1_T212 is identified as a unique phosphorylated site within the PAK subfamily, as it is not conserved across other members of the same family (Molli et al., 2009; Wang and Guo, 2022). Our analysis further validated PAK1_T212 as a unique phosphorylated site based on its conservation score. Moreover, we identified CDK20 as an upstream kinase specifically targeting the unique phosphorylated site T212 in PAK1, establishing CDK20-PAK1 as a unique pathway.

This approach highlights the importance of characterizing distinct phosphorylated sites within kinase families, thereby enhancing our understanding of the kinase-specific regulatory networks of functional significance. These applications enhance the potential for precision medicine in treating kinase-driven diseases. In summary, this comprehensive mapping of conserved and unique phosphorylated sites serve as a valuable resource for therapeutic targeting, enabling broad-spectrum and selective intervention strategies in kinase-related diseases.

### Analysis of the functional phosphorylated sites based on multiple features

**FIGURE 4:**
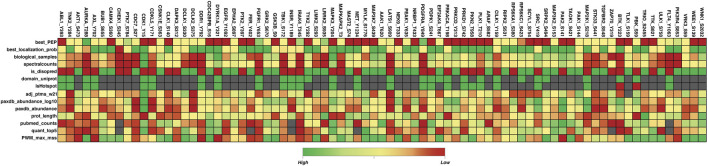
Heatmap of the 80 most dominant phosphorylated sites and their 14 different feature scores reported in the study by Ochoa et al. (2020). This figure represents different feature scores for 80 top predominant phosphorylated sites. Features with the highest, lowest, and empty scores are depicted in green, red, and gray colors respectively. Figure generated using the data given in Ochoa et al. (2020); Only a few features are included here. Best_PEP: Peptide posterior empirical probability (PEP) from MaxQuant; best_localization_prob: MaxQuant localization probability; biological_samples: Number of biological samples in which site was detected by MS; spectralcounts: Number of spectral counts based on MaxQuant; is_disopred: Sequece-based prediction of disorder (disopred); domain_uniprot: Is the residue in an annotated domain (uniprot); isHotspot: is in conserved phosphorylation hotspot; adj_ptms_w21: Number of adjacent Ptms in 21 residue window derived from PhosphoSitePlus; paxdb_abundance_log10: Protein abundance (PaxDb - log10); paxdb_abundance: Protein abundance (PaxDb); prot_length: Protein length; pubmed_counts: Number of publicly available quantitative studies reporting the site; quant_top5: Number of conditions in which the site is quantified within the top 5% differentially regulated sites; PWM_max_mss: Maximum mss score similarity between site flanking region and known kinase position weight matrix (PWM).

Among the total phosphorylated sites identified in the human kinome, 293 phosphorylated sites were identified as the top predominant site or most frequently detected phosphorylated site in each kinase based on their frequency of detection. In 293 top predominant sites, 248 phosphorylated sites possess a functional score according to the study by Ochoa et al. (2020), 45 phosphorylated sites did not possess any functional score according to the previous study. Among the 690 highly conserved phosphorylated sites, 55 possess a functional score. Out of the 1,721 less conserved phosphorylated sites, 107 have been predicted as functional by Ochoa et al. (2020). These results indicate the functional relevance of less conserved phosphorylated sites or unique phosphorylated sites analyzed by our approach. The functional score was generated using machine learning approaches, they considered around 80 different features such as MS supporting evidence, Residue conservation, phosphorylated site age, Consensus motif binding, Conditional phospho-regulation, One-dimensional structural properties, Phosphorylation structural hotspots, Effect on protein stability and interaction interfaces, and Protein topology annotations, etc*.*, relevant to sequence as well as structural level (Figure 4) (Supplementary Table S3)

### Annotation of distribution of predominant phosphorylated sites in human kinome based on the contemporary global phosphoproteome datasets

We annotated the human kinome based on the distribution of predominant phosphorylated sites relative to the kinase domain. This classification moves beyond traditional methods, which focus primarily on kinase domain homology or catalytic activity, by incorporating phosphorylated site positional data to reflect unique regulatory patterns across kinases.

**FIGURE 5:**
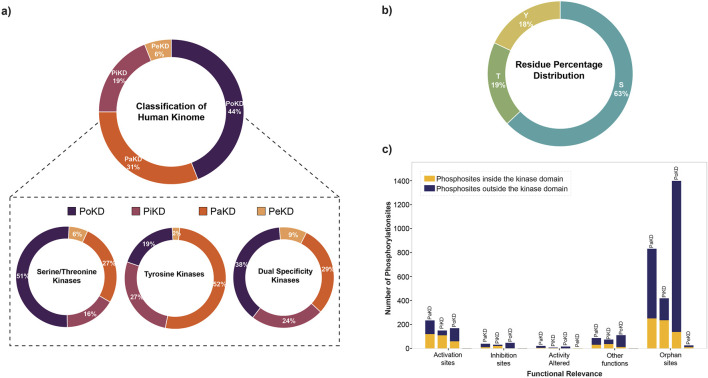
**(a)** Percentage distribution of different categories of human kinome based on predominant phosphorylated sites; **(b)** Percentage distribution of amino acid residues (S/T/Y) identified as predominant phosphorylated sites identified in 449 out of the 519 kinases analyzed; **(c)** Functional relevance of predominant phosphorylated sites in different kinase categories.

**FIGURE 6:**
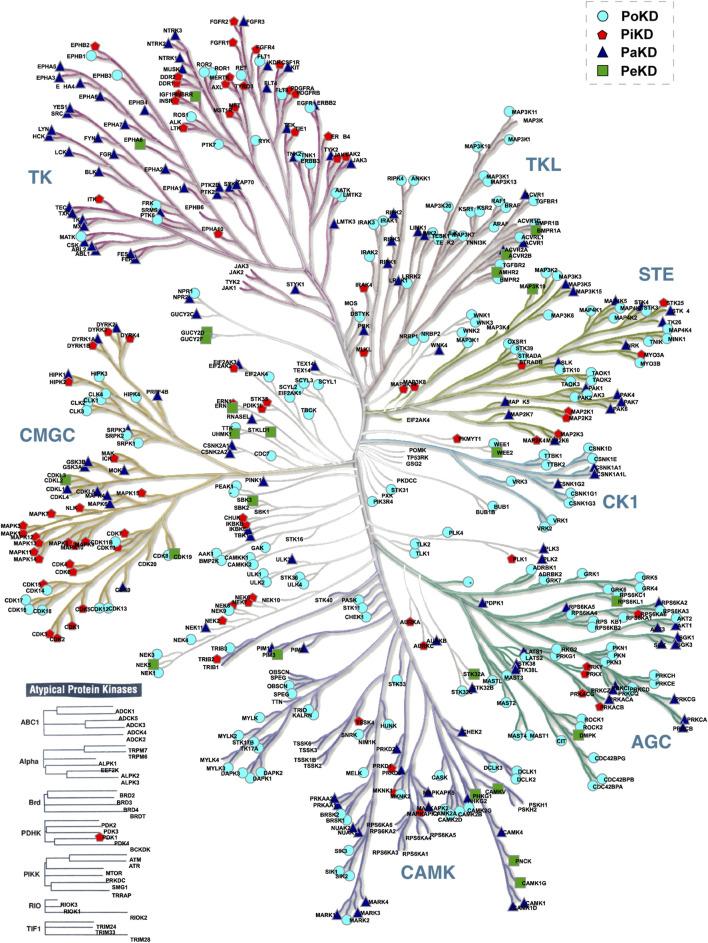
Positioning of the predominantly detected phosphorylated sites in kinases in the human kinome tree.

Based on the predominant or frequently detected phosphorylated sites and their localization in the kinases relative to their kinase domain, we categorized kinome into those with Predominant phosphorylated site in the Kinase domain (PiKD), Predominant phosphorylated site outside the Kinase domain (PoKD), Phosphorylated sites mixed in- and outside Kinase domain is represented as Phosphorylated sites apportioned in Kinase and distributed (PaKD), and, Predominant phosphorylated sites enigmatic to the Kinase and kinase domain (PeKD). The classification mainly focuses on the position of the first three predominant phosphorylated sites, whether inside or outside the protein kinase domain. Kinases with no phosphorylated sites or possessing only one phosphorylated site were grouped under the PeKD category (Figure 5; Supplementary Table S4). Classified kinases were represented in the human kinome tree (Figure 6).

From the analysis of the functional relevance of these phosphorylated sites, we have identified that across all categories, phosphorylation is more frequently detected outside the kinase domain. Activation sites exhibit a moderate number of phosphorylation events, with a relatively balanced distribution across PaKD, PiKD, PoKD, and PeKD. Inhibition and activity-altered sites contain fewer phosphorylation events, with inhibition sites showing a slight preference for kinase-domain phosphorylation. Phosphorylated sites with other functions are also represented in a small number of phosphorylation events, with a slight dominance of kinase-domain phosphorylated sites. Orphan sites, which lack a known function, form the largest category, with PoKD and PaKD representing the highest number of phosphorylation events, predominantly outside the kinase domain. Significantly, PoKD has the highest number of orphan phosphorylated sites, indicating potential regulatory roles that require further investigation. Overall, this analysis highlights the prominence of non-kinase domain phosphorylation, particularly among orphan sites, emphasizing the need for additional functional characterization (Figure 5C).

**FIGURE 7:**
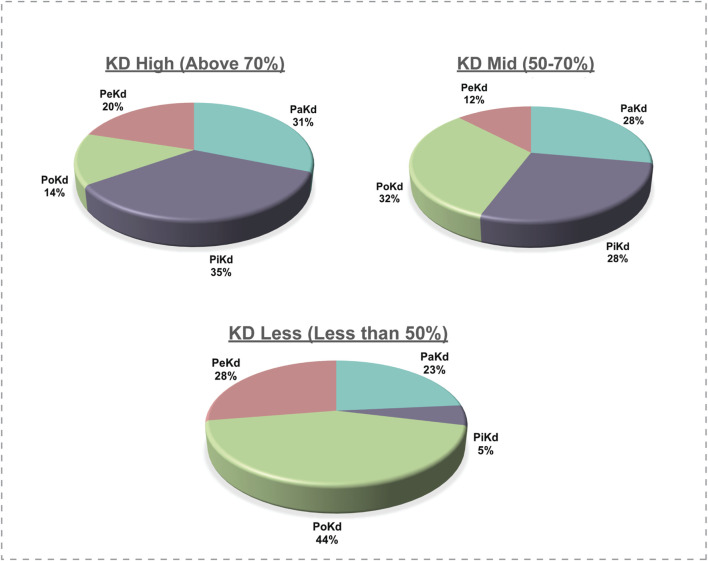
Correlation of phosphorylated site location with the total kinase protein length; KD: Kinase Domain.

Kinases are complex enzymes whose activity is regulated through precise structural organization and allosteric interactions. To fully grasp their function and regulation, it's important to study them in their complete, full-length form (Reinhardt and Leonard, 2023). Analysis of the protein kinase domain in each kinase revealed that the kinase domains vary significantly in length between 3% and 97% of the total protein’s sequence across families (Supplementary Table S5). Based on the length of the protein kinase domain over the total sequence length (kinase-domain-length/kinase-length ratio), we have classified the kinases into three categories as “KD High”, “KD Mid” and “KD Less”. The “KD High’ group consists of the kinases with a kinase domain of 70% and “KD Mid” contains Kinases with 50%–70% kinase domain, whereas “KD Less” contains less than 50% of the kinase domain across the sequence (Figure 7). This led to reveal the PoKD kinases such as BMPR1B, TGFBR1, ACVRL1, FLT3, TGFBR2, RYK, VRK1, STRADA, DAPK2, EPHB1, EPHB3, and RPS6KA1, etc*.*, with >70% (kinase-domain-length/kinase-length ratio) and PiKD kinases such as MYO3A, MAP3K14, ERN1, HIPK2, PRKD1, MAPK7, NEK8, IKBKB, and CHUK with <30% ratio (Supplementary Figure S3). Examining the 293 top phosphorylated sites, which represent the most frequently detected site in each kinase, uncovered that 6% were found within disordered regions.

**FIGURE 8:**
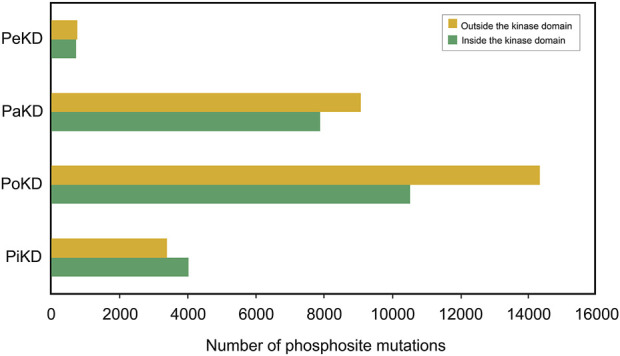
Phosphorylated site sequence variations in different category of kinases.

Our analysis indicates that most phosphorylated site mutations are located outside the kinase domain. In the PoKD, PaKD, and PeKD categories, a majority of the phosphorylated sites situated outside the kinase domain exhibit sequence variations. While phosphorylated site sequence variations in PiKD kinases are more prevalent within the kinase domain, the difference in variation frequency between sites inside and outside the kinase domain is relatively small (Figure 8; Supplementary Table S5). Notably, all these variations have been reported to be associated with various cancers. These findings highlight the significant role of phosphorylated sites located outside the kinase domain and may contribute to the disease mechanisms. Hence our classification of kinases based on the positional distribution of predominant phosphorylated sites relative to their kinase domain opens up new avenues and hypothesis-driven approaches to analyze the structure and function of kinases based on their predominant sites for targeted therapeutic approaches.

Highlighting these predominant phosphorylated sites is not intended to nullify the impact of less-predominantly detected or dynamically regulated phosphorylated sites. Rather these phosphopeptides may be high-flying in mass spectrometry and could also be an outcome of the current phosphoproteomics platforms/pipelines. Either way, these predominant phosphorylated sites would continue to be detected using phosphoproteomics. Hence, the orphans among them demand targeted studies to evaluate their role in the kinase activity or protein stability for their accountability and enhancement of phosphoproteome data analysis. The kinase-independent functions of kinases are largely being established (Rauch et al., 2011) and for many of them, their contribution to carcinogenesis is also reported. In light of these findings, the current study also enlightens the potential for phosphorylated site-directed therapeutic approaches on kinase beyond the kinase-activity dependent cancer therapy.

Our analysis classified TAOK1 as Predominant phosphorylated site outside the Kinase domain (PoKD) kinase due to distribution of its predominant phosphorylated sites (S421, S9, S965 and S445, *etc.*) were situated non-kinase domain regions. In our previous study, we identified the functional roles of these predominant phosphorylated sites. We proposed S9 as an autophosphorylation site and S421 as TAOK1 kinase activity-associated phosphorylated site involved in the maintenance of genome integrity (Priyanka et al., 2024). Such analyses showed the potential relevance of phosphorylated site positions and our classification framework highlights the phosphorylated sites that may act as molecular switches in both kinase and non-kinase regions.

**FIGURE 9:**
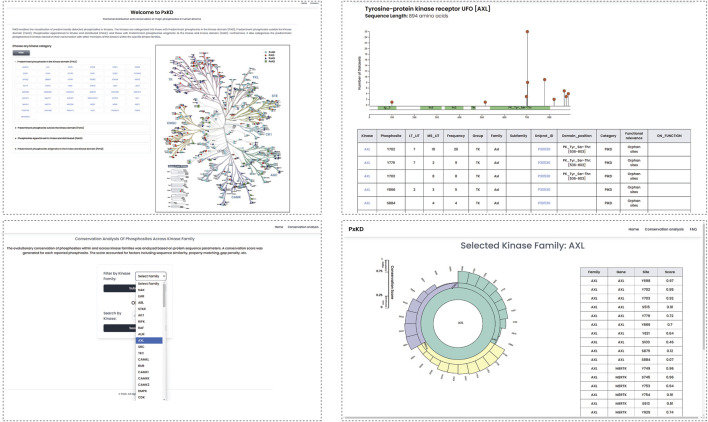
The PxKD database facilitates the visualization of predominant phosphorylated sites identified in each kinase and the classification of human kinome based on predominant phosphorylated sites.

We created a resource PxKD Phosphorylated sites [Predominant-Phosphorylated site x (classified) in Kinases based on their Distribution in primary sequence] with reference to the kinase domain for visualization of their representative phosphorylated sites. PxKD can be accessed through the link (https://ciods.in/pxkd/) (Figure 9). Through PxKD we can analyse the conservation of phosphorylated sites across kinase families with a search by kinase or by kinase family name. It will include the sequence alignment of the phosphomotifs across the kinase family.

### Potential applications of the functional characterization of predominant Kinase phosphorylated sites

The comprehensive characterization of human kinases based on their representative phosphorylated sites provide a foundation for addressing key phosphoproteomics and kinase biology demands. It enables the systematic identification and mapping of predominant/representative phosphorylated sites, offering insights into their critical roles in kinase activity and regulation. This study also emphasizes the importance of elucidating the functional roles of these sites, and addressing knowledge gaps in phosphorylation-dependent processes. Additionally, it highlights the presence of orphan-predominant phosphorylated sites, those without known functional associations, underscoring the need for further investigation and prioritization to explore their potential biological significance, including their roles in allosteric regulation.

This characterization would contribute to exploring the roles and functions of kinases in diverse biological conditions as a reference point to relate to other phosphorylated sites, especially to predict their role in interaction with other proteins or their substrate specificities. Moreover, these findings can be directly integrated into phosphoproteome enrichment analysis tools, with predominant phosphorylated sites serving as representative markers of kinase activity or functions. This integration would enhance the precision of enrichment analyses to enable more insightful interpretations of large-scale phosphoproteomics datasets and drive further advancements in systems biology and therapeutic research.

## Discussion

Our study presents a comprehensive analysis of phosphorylated sites in the human kinome, offering new insights into kinase regulation, diversity, and therapeutic implications through a phosphorylated site-centric framework. By examining the positional distribution of predominant phosphorylated sites, either within or outside the kinase domain, we address a critical gap in kinase classification and functional analysis. Traditional approaches often emphasize domain homology and sequence similarity; however, these methods lack the granularity to capture the regulatory significance of phosphorylation sites within and outside the kinase domain. Our findings suggest that a nuanced classification of predominant phosphorylated sites detected in kinases based on phosphorylated site positioning is essential for a deeper understanding of kinase regulatory networks and functional heterogeneity in the future.

With approximately 75% of phosphorylated sites in kinases reported through high-throughput (HT) methods, a significant challenge is the experimental validation of the majorly detected phosphorylated sites. High-confidence phosphorylated sites often require confirmation through low-throughput (LT) methods, such as targeted kinase assays, which provide robust evidence for individual phosphorylation events but lack the scalability of HT approaches. By integrating data from HT and LT sources, we established a comprehensive dataset that allowed us to rank phosphorylated sites by detection frequency and classify kinases based on the positional distribution of predominant phosphorylated sites.

Our investigation into conserved and non- or partially-conserved unique phosphorylated sites across kinase families reveals significant insights into kinase-specific regulatory mechanisms. Conserved phosphorylated sites across families appear to be central to core catalytic and regulatory functions, making them valuable targets for broad-spectrum therapies. In contrast, unique phosphorylated sites, which may be involved in kinase-specific regulatory functions, offer the potential for more selective therapeutic strategies, potentially reducing off-target effects associated with conventional broad-spectrum kinase inhibitors. The finding that many frequently detected phosphorylated sites in kinases associated with dysregulation in diseases are located outside the kinase domain, suggests unexplored regulatory roles and new therapeutic opportunities. This challenges the traditional focus on kinase-domain phosphorylation sites and opens avenues for kinase-targeted therapies that consider regulatory phosphorylation events beyond the active site.

## Conclusion

In summary, our phosphorylated site-based classification of kinases would contribute to enhancement in the understanding of kinase regulations at structural and functional context. It offers valuable insights for advanced kinase-targeted therapies employing phosphorylated site-directed therapies. By highlighting the importance of predominant phosphorylated sites, this framework contributes to a refined view of kinase functionality and specificity. This approach not only holds the potential for improving the precision of kinase-targeted interventions but also underscores the therapeutic promise of targeting regulatory phosphorylated sites to achieve selective modulation of kinase activity in diverse disease contexts. In conclusion, this study aims to propose a phosphorylated site-centric analysis of human kinases and their functional relevances.

## Data Availability

The datasets presented in this study can be found in online repositories. The names of the repository/repositories and accession number(s) can be found in the article/Supplementary Material.

## Supplementary material

SUPPLEMENTARY FIGURE S1 In silico digestion of phosphopeptides corresponding to the phosphorylated sites identified through low-throughput experiments (LT_only).

SUPPLEMENTARY FIGURE S2 Kinase-kinase interactions specific to uniquely phosphorylated sites. Activation sites were represented in green colour. Inhibition sites were represented red colour.

SUPPLEMENTARY FIGURE S3 Predominant phosphorylated sites represented across kinase sequence.

SUPPLEMENTARY TABLE S1 Kinome phosphorylated sites reported in PhosphoSitePlus database.

SUPPLEMENTARY TABLE S2 Kinase family-based conservation of phosphorylated sites reported in kinases.

SUPPLEMENTARY TABLE S3 Functional phosphorylated sites identified among the top predominant phosphorylated sites.

SUPPLEMENTARY TABLE S4 Classification of human kinome based on predominant phosphorylated sites.

SUPPLEMENTARY TABLE S5 Classification of kinases based on the length of kinase domain over sequence length.
