# Supplementary figures and images for: Positional distribution and conservation of major phosphorylated sites in the human kinome
Source: Front Mol Biosci. 2025 Apr 9;12:1557835. doi: 10.3389/fmolb.2025.1557835 (PMC12015135; doi:10.3389/fmolb.2025.1557835)

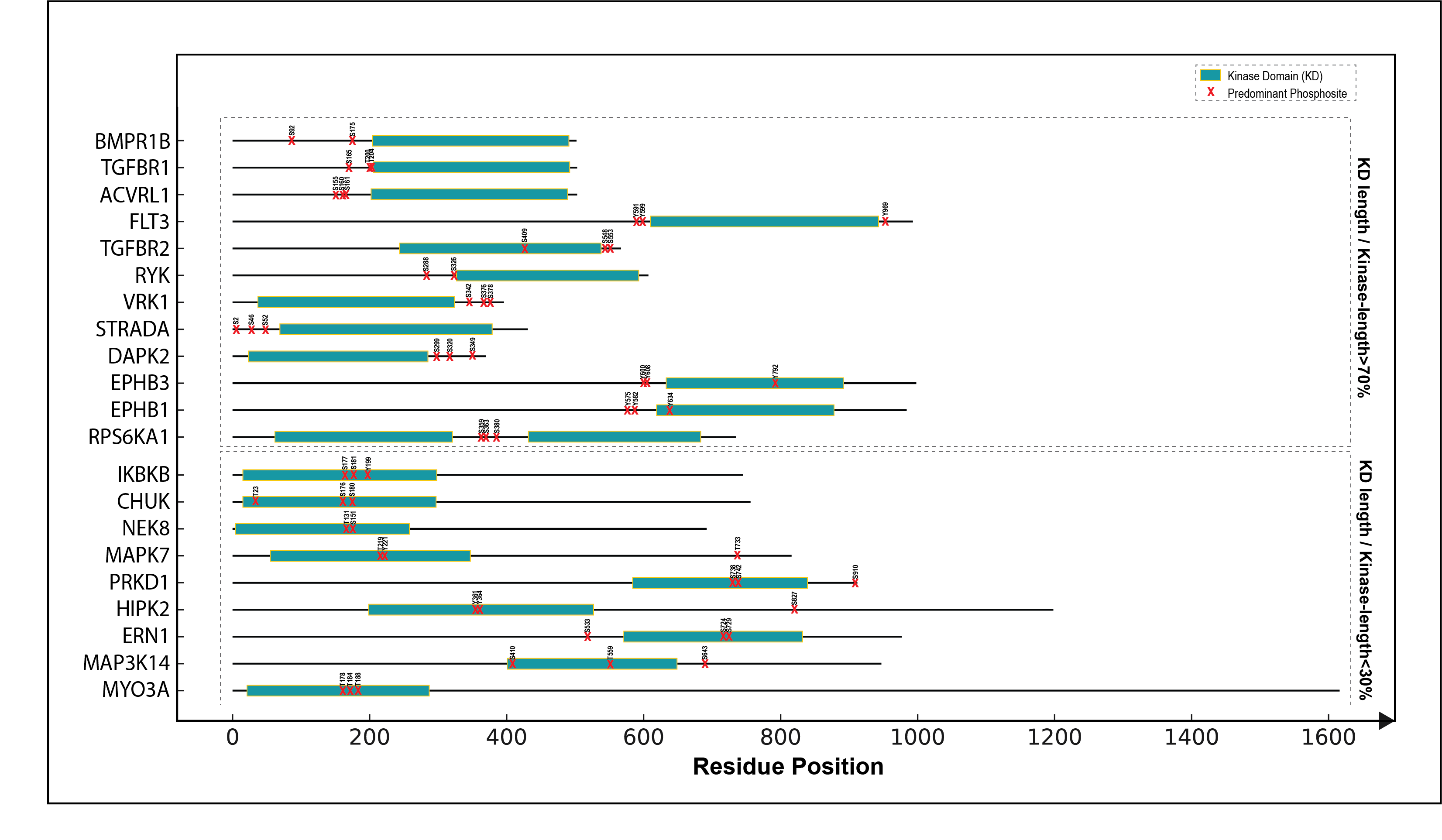

Supplement: Supplementary file 3 [file Image3.tif]

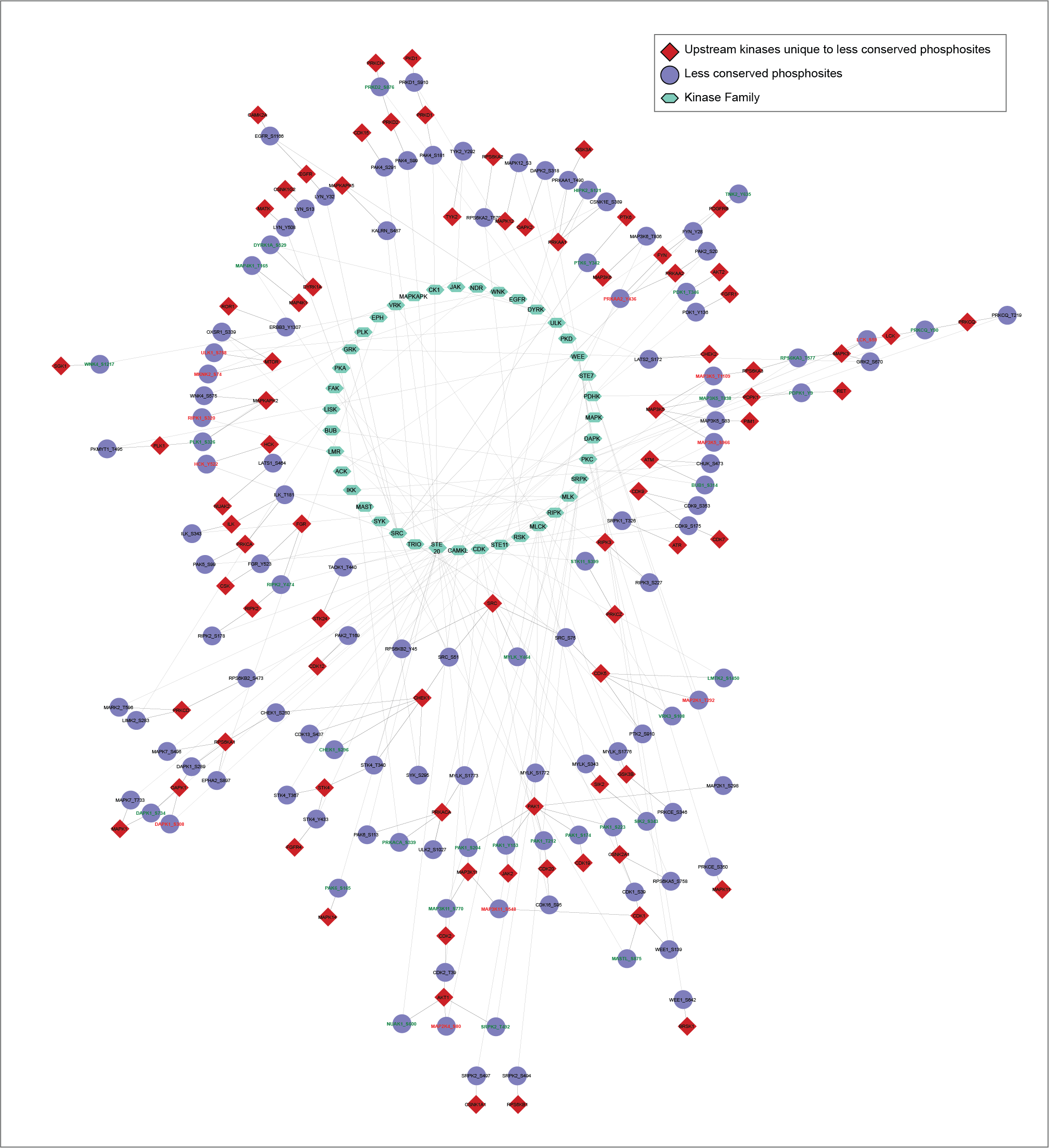

Supplement: Supplementary file 4 [file Image2.tif]

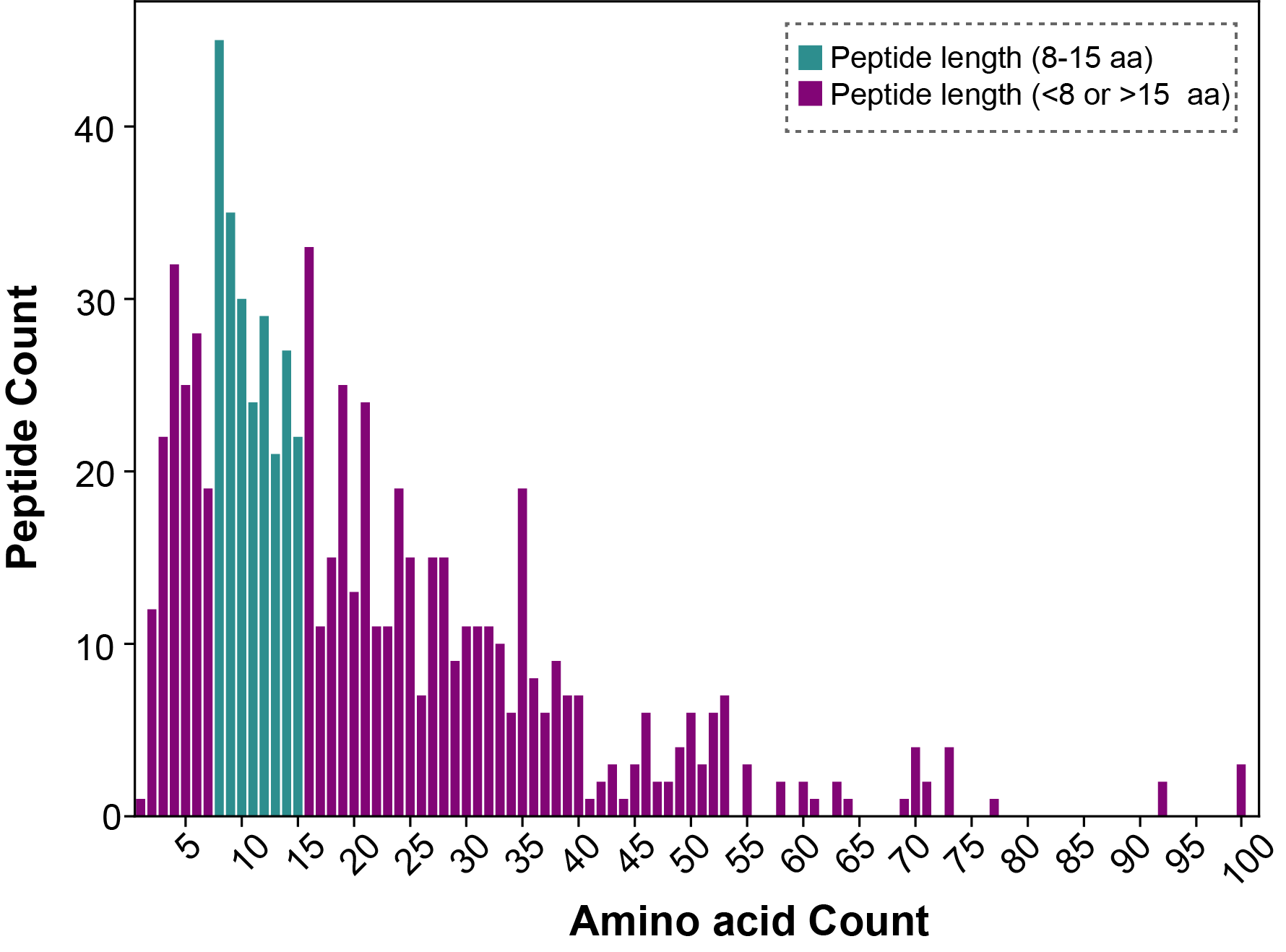

Supplement: Supplementary file 5 [file Image1.tif]
